# Objectivation of laryngeal electromyography (LEMG) data: turn number vs. qualitative analysis

**DOI:** 10.1007/s00405-020-05846-7

**Published:** 2020-02-18

**Authors:** Lukas Kneisz, Gerd Fabian Volk, Winfried Mayr, Matthias Leonhard, Claus Pototschnig, Berit Schneider-Stickler

**Affiliations:** 1 Center for Medical Physics and Biomedical Engineering, Medical University of Vienna, Währinger Gürtel 18-20, 1090 Vienna, Austria; 2 Department of Otorhinolaryngology, Jena University Hospital, Am Klinikum 1, 07747 Jena, Germany; 3 Division of Phoniatrics-Logopedics, Department of Otorhinolaryngology, Medical University of Vienna, Währinger Gürtel 18-20, 1090 Vienna, Austria; 4 Department of Otorhinolaryngology, Medical University of Innsbruck, Anichstrasse 35, 6020 Innsbruck, Austria

**Keywords:** Vocal fold immobility, Laryngeal electromyography, Quantitative electromyography, Laryngeal paresis, Turns analysis

## Abstract

**Purpose:**

This paper describes a first attempt to quantify LEMG data based on turn number calculation. The results obtained for both healthy and ailing thyroarytenoid (TA) muscles of patients with unilateral vocal fold immobility (UVFI) were compared with the respective qualitative evaluation concerning volitional activity to determine whether the two types of analyses deliver similar results.

**Methods:**

LEMG data obtained from 44 adults with UVFI were considered for the study. Semiquantitative evaluation of TA volitional activity and turn number were assessed for the ailing and the healthy TA and the difference in percentage was calculated. Paired data were compared with the Wilcoxon signed-rank test. The volitional activity assessment and the turn number evaluation were compared with the Kruskal–Wallis test, and their relationship was tested with the Kendall rank correlation.

**Results:**

Datasets of 27 patients were considered compatible with turns/s calculation. The results showed that complete paralysis correlated with no turns; single fiber volitional activity with 62–208 turns/s, strongly decreased volitional activity with 198–501 turns/s; and dense volitional activity with 441–1234 turns/s. On the ailing VF only, the Kruskal–Wallis test showed a statistically significant difference (*p* = 0.0001), and the Kendall rank correlation a positive relationship (*r* = 0.853*,p* ≤ 0.0001) between the volitional activity rating and the turn number assessment.

**Conclusions:**

Our preliminary results showed that turn number evaluation is an effective tool to confirm LEMG qualitative analysis, and that, in combination with laryngostroboscopy and voice assessment, can help improving the accuracy of the diagnosis and prognosis and the effectiveness of the chosen therapy.

## Introduction

Electromyography (EMG) is a diagnostic procedure to assess the status of muscles and their innervating motor neurons. It is used to discriminate nerve vs. muscle dysfunctions and to detect defects in the nerve-to-muscle signal transmission [1]. The first laryngeal EMG (LEMG) was performed by Weddell [2]. Since then, while recognized as promising diagnostic tool in the worldwide ENT community [3], LEMG has been struggling to become a routinely used diagnostic technique. The major drawbacks of LEMG are on one side the lack of a standardized procedural protocol, and on the other side the fact that its results are qualitative rather than quantitative, completely based on the subjective interpretation of experts in the field [4]. These characteristics make this type of analysis suitable only for a limited group of ENT doctors with decades of experience with this procedure and its interpretation. To overcome these difficulties, in 2008, a group of European expert neurolaryngologists founded a working group aiming to provide systematic guidelines for LEMG execution and analysis. In 2012, the group published a detailed guideline on (1) the minimum equipment requirements for performing and recording LEMGs; (2) the procedural steps to guarantee reproducible results; (3) the essential LEMG interpretation criteria to ensure result standardization, and (4) launched the website https://www.lemg.org to encourage experience exchange among the sites regularly performing LEMG and those still struggling with its implementation in their routine protocol. The European Laryngological Society emphasized the need of standardized training and regular practice [5] particularly considering the initial results of the international LEMG registry conducted by this working group between 2012 and 2016, aiming to evaluate the implementation of the LEMG across Central and Eastern Europe. The registry results clearly showed the limit of qualitative analysis. To our best knowledge, there is no standardized protocol available to effectively quantify the LEMG results.

The results presented in this work aim to evaluate a preliminary protocol for the quantification of the LEMG results and to compare them with a typical qualitative analysis. This latter type of analysis is mainly performed by following the guideline published by Volk et al. [4]. On the contrary, the quantification of LEMG results requires a good understanding of the signal characteristics. In short, any EMG signal can be evaluated in time (i.e. variations of amplitude with time) or in frequency (i.e. number of times an event occurs in the total period of observation). A quantification of the signal can be achieved through the evaluation of “turns” per second, where “turns” are defined as any 100 µV signal amplitude change in the rising or dropping slope of the LEMG signal. Provided that the slope extremities do not overlap, this type of analysis allows an easy exclusion of false positives linked to the presence of interferences or background noises. In 1991, McGill et al. [6] first described a significant correlation between pathologically increased [7] morphologic Motor Unit Action Potentials (MUAPs) and decreased number of turns in neuropathic lesions. Later studies confirmed that lesions of the lower motor neurons innervating voluntary muscles severely impair the motor unit recruitment pattern while increasing the MUAP [7]. Later studies were able to roughly identify the typical number of turns/s expected for various healthy muscles, including the laryngeal ones [8–10]. Based on all these previous results, we developed a protocol to count the turn number from the respective LEMG for the healthy and ailing TA muscle of patients suffering from unilateral vocal fold immobility (UVFI). We then compared these results with a qualitative analysis of the volitional activity of the same muscle to determine a correlation between the two types of analyses and determine whether their combination would provide a more accurate diagnosis, prognosis, and consequent improved therapeutic approach for the patients.

## Methods

### Registry characteristics

The data presented in this article were generated between October 2012 and December 2014 by the Medical University of Vienna within an international, observational, non-interventional, systematic registry with both retrospective data collection and prospective patient recruitment. The registry was approved by the ethics committee in 2013 (application number 1524/2013).

### Population

44 adult subjects of both sexes were recruited, who were diagnosed with UVFI by means of anamnestic evaluation and laryngoscopy, between 4 weeks and 10 years before undergoing the LEMG.

### LEMG performance

The LEMG measurements were conducted according to [4] with disposable concentric needle electrodes with a recording area of 0.07 mm^2^ (TECA Elite, CareFusion, Middleton, WI, USA), connected to the VikingQuest^®^ electromyography acquisition system (Natus Medical Incorporated, Pleasanton, CA, USA). In short, the patient was asked to sit upright on an examination chair. A unidirectional microphone was mounted 20 cm from the patient’s mouth. The voice signals were recorded time-synchronous with the electromyography acquisition system. A needle electrode was inserted in the healthy thyroarytenoid muscle (TA) and the patient was first asked to phonate [a:] at habitual pitch and comfortable loudness for at least 3 s and then to complete forced sniffing inspiration three times, in order to confirm the correct identification of the TA muscle [9], and to diagnose the presence of synkinetic (misdirected innervation of a damaged recurrent laryngeal nerve fibers to VF abductor and adductor muscles) reinnervation. The same maneuvers were then completed on the ailing side. The amplitude resolution of the EMG signal was set to 100 µV/division. EMG signal with voltages higher than 1 mV appear clipped, because the amplifier drives into saturation. In such a case the affected sequences were repeated at 200 µV/division, unless it caused discomfort to the patient.

### LEMG qualitative interpretation

LEMG qualitative interpretation was completed by a team of experts, immediately after the conclusion of the respective measurement, based on the characteristics of the EMG waveforms (high-pass filter set to 20 Hz, low-pass filter setting 10 kHz, sampling frequency set to 20 kHz). Epochs of 200 ms were displayed on-screen by setting the sweep speed to 10 ms/division. The volitional activity was scored according to [4].

### Turns/s calculation

**Fig. 1:**
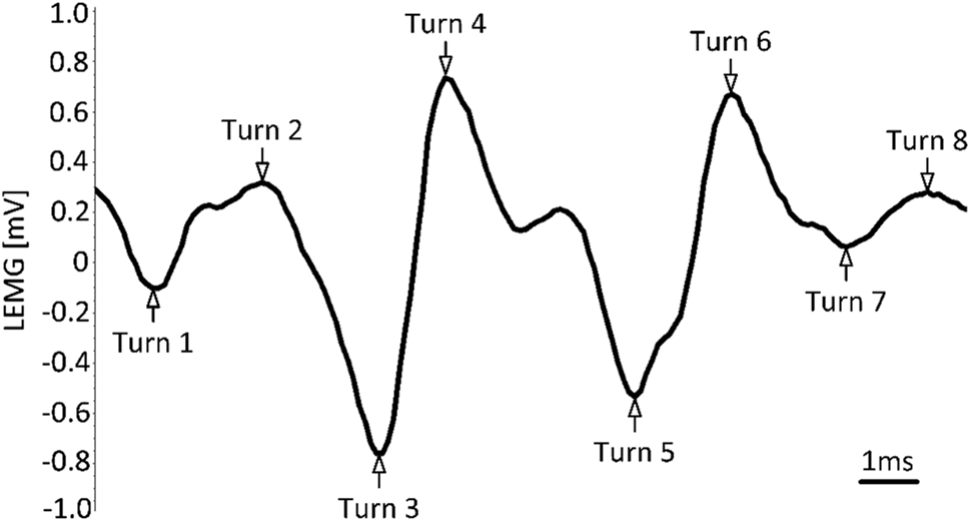
Example of a turns/s calculation in a 15 ms LEMG segment. The total number of detected turns was eight, since the peak observed between Turn 4 and Turn 5 was below the threshold of 100 µV and therefore excluded from the calculation

**Fig. 2:**
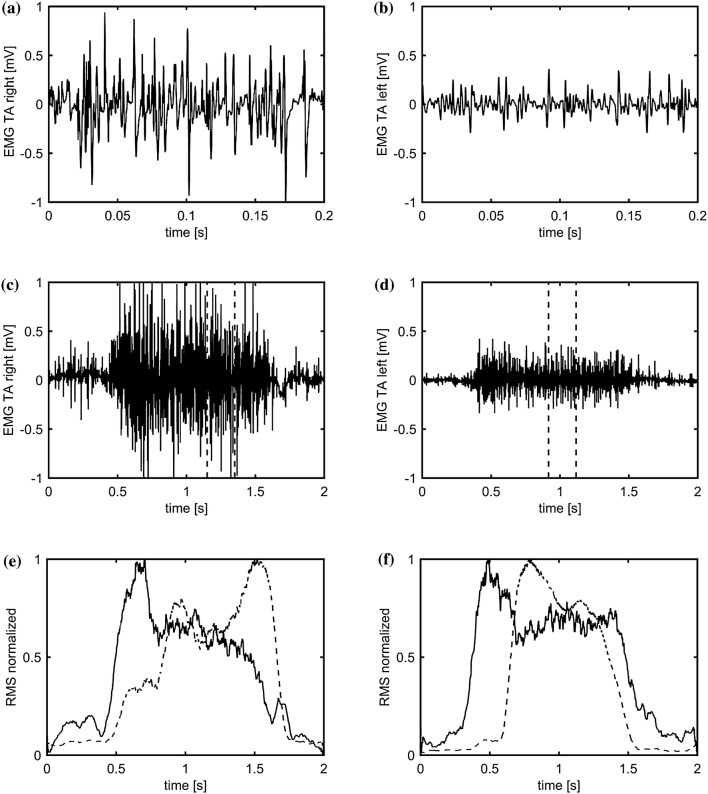
Turns/s evaluation for the healthy (right) and ailing (left) VF of the subject LERR021217. **a**, **b** 200 ms section of the TA EMG with 10 ms/division time-base. **c**, **d** 2 s section of the TA EMG with 100 ms/division time-base during phonation. Vertical dotted lines indicate the 200 ms portion showed in **a**, **b**, respectively. **e**, **f** 2 s section of the normalized root-mean-squared (RMS) envelopes which are calculated as the integral of the squares of the microphone signal (dotted line) and the TA EMG (black line) showing the onset and intensity of phonation (dotted line) and the corresponding onset and strength of the EMG activity (black line)

Turns/s calculation was completed offline by a team of experts by means of the built-in algorithm of the Viking Nicolet software (VikingQuest 12.1). Datasets in which the needle electrode could not be placed in the target muscle, or in which the maximum EMG amplitude of the volitional activity was below 100 µV, were excluded from turns analysis. Datasets in which the activity of the targeted TA muscle was found higher between two consecutive phonations than during phonation itself and incompatible with synkinetic reinnervation were likewise excluded to limit the effects of artefacts on the turns/s analysis. The presence of spontaneous motor unit activity, or phonation segments shorter than 3 s were considered exclusion criteria. In short, the number of peaks in the analysis window above the threshold of 100 µV was calculated and normalized to an equivalent time-base of one second (turns/s), as depicted in the example reported in Fig. 1. In short, a segment for analysis of 500 ms was identified with the help of the time-synchronous microphone signal. Turns/s scores, relative to each of the three times when the patient was asked to phonate [a:], were averaged for each evaluated TA muscle (see Fig. 2). To determine the difference in turns/s scoring between the ailing and the healthy TA, the following formula was used:

$${\text{Percentage decrease}} = \frac{{{\text{turns/s affected side}} - {\text{turns/s healthy side}}}}{{\text{turns/s healthy side}}} \times 100.$$

### Statistical analysis

Patient demographics and outcome variables were analyzed with IBM SPSS statistics software (Version 25; IBM, New York) for medical statistics. Descriptive statistics were used to report demographic data (e.g. age, gender). Quantitative data are presented as mean, standard deviation (SD), range (minimum and maximum), median, and interquartile range (IQR); qualitative data are presented as absolute and relative frequencies. The distribution of turns/s samples was tested by the Kolmogorov–Smirnov test for normality, mean values and standard deviations, median, and IQR, and compared with the results of the qualitative analysis of the volitional activity according to the following rating scale: 4 = dense, 3 = mildly decreased, 2 = strongly decreased, 1 = single fiber, 0 = no activity. Paired data were compared with the Wilcoxon signed-rank test. Kruskal–Wallis test was used to determine whether the results of the qualitative analysis (volitional activity) match or differ from the turn number evaluation. The Kendall rank correlation was performed to determinate whether a relationship exists between volitional activity and the turn number assessment.

## Results

Datasets of 27 patients were considered compatible with turns/s calculation. 16/27 (59.3%) patients were male and 11/27 (40.7%) were female. The average age was 57.2 ± 15.6 with patients between 18 and 80 years. Details about the UVFI etiology and duration until the time the EMG was performed are provided in Tables 1 and 2.

**Table 1:** UVFI etiology

Iatrogenic	19/27 (70.4%)
Traumatic (non-surgical-trauma)	2/27 (7.4%)
Idiopathic	4/27 (14.8%)
Cancer	2/27 (7.4%)

**Table 2:** UVFI duration at the time of the EMG in months (mo)

Duration of the UVFI	No. of patients
≥ 1 months < 3 months	9/27 (33.3%)
≥ 3 months < 6 months	5/27 (18.5%)
≥ 6 months < 12 months	5/27 (18.5%)
≥ 12 months	7/27 (25.9%)
Unknown	1/27 (3.7%)

Turns/s could be calculated in 27/27 (100%) healthy and in 20/27 (74.1%) of the ailing TA muscles. In 7/27 (25.9%) of the ailing TA muscles, the resulting amplitude resolution of the EMG signal was below the threshold and therefore could not be used for analysis. In 6/20 ailing TA muscles EMG activity was absent resulting in 0 turns. The distribution of the turns in both cases showed a non-Gaussian shape according to the one-sample Kolmogorov–Smirnov Test (*p* ≤ 0.05).

The average number of turn/s characterizing the ailing VFs was 210 ± 264, while on average the turn number of the healthy VFs was 720 ± 297. The Wilcoxon Signed Rank Test showed that this difference in this pattern of turns/s distribution between the ailing and the healthy VF was significant (*p* ≤ 0.0001). The qualitative analysis performed according to [4] showed that the volitional activity was found normal (i.e. dense) in the majority of the healthy (75%), while it was absent (30%) or decreased (70%) in all the analyzed ailing VFs.

**Fig. 3:**
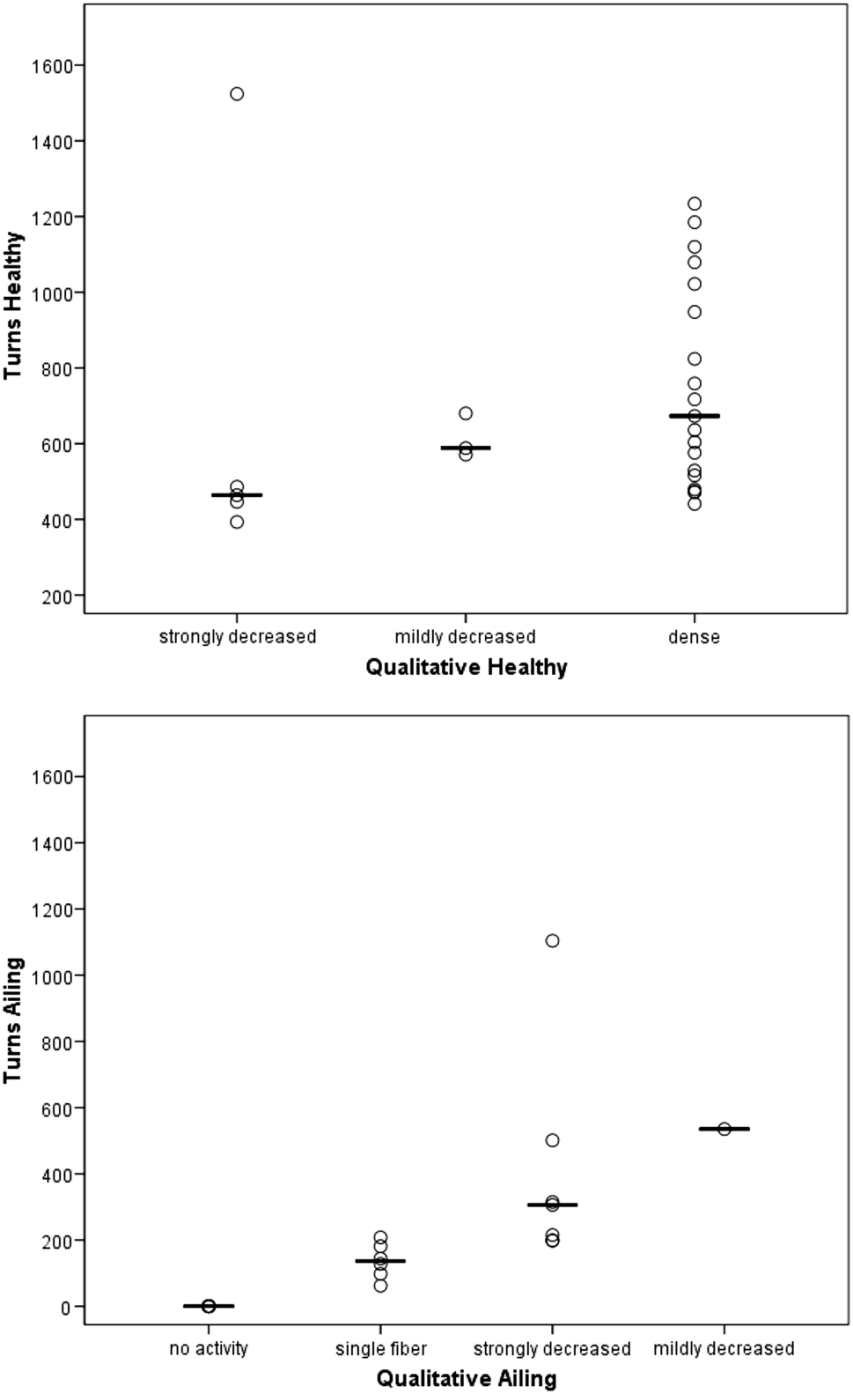
**a** The dense volitional activity predominant in healthy TAs corresponds to a high number of turns. **b** The decreased to absent volitional activity predominant in ailing TAs corresponds to a low number of turns

The Kruskal–Wallis Test showed that there is a statistically significant difference between the volitional activity rating and the turn number assessment performed on the ailing (*p* = 0.001), but not on the healthy TA muscles (*p* = 0.226). The Kendall rank correlation showed a strong, positive relationship between the volitional activity rating and the turn number assessment on the ailing (*r* = 0.853*,p* ≤ 0.0001), but not on the healthy TA muscles (*r* = 0.268*,p* = 0.089). The relationship between the turn number and qualitative results of the volitional activity evaluation is depicted in detail in Fig. 3a, b, respectively.

In 20/27 (74.1%) of the evaluated subjects both the ailing and the healthy VFs were evaluated in the same session. In the remaining 7/27 (25.9%) subjects, the resulting amplitude resolution of the EMG signal of the ailing VF was below the threshold and therefore could not be used for analysis. The results of the qualitative and quantitative analysis are summarized in Table 3. While, as expected the complete lack of volitional activity correlated 100% with no turns, the classification of the volitional activity as normal (or dense) correlated with a turn number comprised between 441 and 1234 with a median of 673 and IQR of 432. Mildly decreased volitional activity correlated with a median number of turns/s of 553. Strongly decreased volitional activity correlated with a turn number comprised between 198 and 1524 with a median of 420 and an IQR of 260. Single fiber volitional activity correlated with a turn number comprised between 62 and 208 with a median of 136 and an IQR of 99.

**Table 3:** Volitional activity score and turn number of the subjects in whom both VFs were evaluated in the same EMG session

Subject ID	Healthy VF		Ailing VF		Decreased turn number in percentage
	Volitional activity	Turn/s	Volitional activity	Turn/s	
LERP0212007	4	1234	0	0	100
LERP0212013	4	948	0	0	100
LERP0212004	4	673	0	0	100
LERP0212022	4	759	0	0	100
LERR0212005	4	473	0	0	100
LERR0212028	4	636	0	0	100
LERP0212006	4	529	1	62	88
LERP0212015	4	604	1	128	79
LERP0212017	4	479	1	98	80
LERR0212027	4	516	1	208	60
LERR0212013	4	1079	1	181	83
LERR0212006	4	1120	2	501	55
LERR0212009	4	441	2	215	51
LERR0212018	4	717	2	199	72
LERR0212017	4	824	3	535	35
LERP0212014	3	571	1	144	75
LERR0212022	2	1524	2	1104	28
LERP0212024	2	464	2	306	34
LERR0212030	2	393	2	315	20
LERR0212032	2	486	2	198	59

In 1/20 (5%) case for which the UVFI had an idiopathic case, the qualitative analysis rated the healthy VF volitional activity mildly decreased. In this case, these results were confirmed by the turns/s analysis.

Considering only the ailing VFs, single fiber volitional activity correlated with a very low number of turns/s comprised between 62 and 208, while strongly decreased volitional activity correlated with an increased number of turns/s comprised between 198 and 501 (if excluding the stenosis case).

As expected, the percentage decrease in turn numbers when comparing the healthy with the ailing VF is 100% when the latter is completely paralyzed. Single fiber volitional activity correlates with a turn number decrease comprised between 60 and 88%. Strongly decreased volitional activity associates with a turn number decrease comprised between 51 and 72% and mildly decreased volitional activity with a turn number decrease of 35%.

## Discussion

Our preliminary data confirmed previous works describing the possibility to use turn number count to objectify the LEMG results. Statham et al. [11] already showed a statistically significant turn number decrease (290 vs. 450) in ailing VF of UVFI patients compared to the healthy one, strongly suggesting that neurogenic damages are well characterized by a low number of turns. Indeed, when we examined a patient with ankylosis and no neurogenic damage, the turn number evaluation rated both the ailing and the healthy VF as normal. Accordingly, in 4/20 (20%) cases, the qualitative analysis rated the healthy VF volitional activity strongly decreased. In 3/4 cases (75%), these results were confirmed by the turn number calculation. In all the 3 cases the etiology (laryngeal tumor, long-term tracheostomy and aortic arch surgery) was compatible with bilateral damage of both VFs. In the other case, the patient was diagnosed with UVFI due to glottic stenosis. In this case, it is expected that while the voice quality is compromised due to impaired glottic closure, the turn number remains unaffected and thus scores as healthy (1524).

Smith et al. [12] reported an average turn number ≤ 400 as pathological. Statham [8] as well as Lindestad [9] suggested to use a lower threshold comprised between 200 and 300 turns/s to discriminate healthy from ailing VFs. While these studies focused on the mere distinction between healthy and ailing, we tried to determine whether it would be possible to use the turn number to categorize objectively and in more detail the grade of damage suffered by the ailing VF, using a similar setting to a previously published LEMG qualitative analysis [4]. Our results showed that both the evaluation of the absolute number of turns or the evaluation of the percentage decreased compared to the contralateral healthy VF, are accurate methods to objectify the LEMG outcomes. The preliminary results of our study, although based on a limited sample of data sets, seem to suggest that dense volitional activity associates with a number of turns/s ≥ 600; mildly decreased volitional activity with a number of turns/s between 400 and 600; strongly decreased volitional activity with a number of turns/s between 200 and 400; and a single fiber volitional activity with a number of turns/s below 200. In a similar way, the decreased turn number for mildly decreased volitional activity is ≤ 35%, for strongly decreased volitional activity is between 50 and 70%, for single fiber volitional activity between 60 and 90%. Both phonation segments shorter than 3 s, which may not be sufficiently long to represent a real situation, and spontaneous motor unit activity were excluded from analysis. Fibrillation potentials with amplitudes around 100 µV were not recorded during phonation. These strict criteria for results analysis were important to increase the internal validity of the study but may have impacted the generalizability of the findings. In all cases, result verification in larger cohort studies are planned.

Ho et al. [13] showed acceptable interrater agreement between 7 experienced raters on LEMG datasets, but 14.29% of rater pairs achieved only fair agreement in the category “strongly decreased recruitment pattern”. The interrater reliability might be improved by providing the number of turns/s in addition to the LEMG signal traces.

Another clinically relevant aspect linked to the turn number calculation, is the better understanding and interpretation of qualitative LEMG results. In particular, in 6/27 cases (22%), the qualitative volitional activity of the healthy VF was rated dense, i.e. normal, while the turn number calculated was comprised between 441 and 529 suggesting a mild decrease in the VF functionality. The VFI etiology for these cases was total thyroidectomy, mediastinoscopy or lung transplantation, i.e. an iatrogenic cause that in principle could have affected both VFs, with the less affected VF being asymptomatic at the time of the LEMG. Since 4/6 cases (66.7%) were evaluated between 1 and 4 months upon UVFI onset, this piece of information would help the medical staff to schedule the upcoming control visits and plan the conservative therapy for the patient as well as to formulate a more accurate prognosis. In this study, we did not focus on the MUAP morphology, which has been previously introduced by other authors to discriminate between healthy and ailing VFs [14, 15]. While this evaluation could become the subject of future studies, its current setting often associates with the detection of false positive results. In particular, it has been reported that the detection of increasing polyphasic MUAPs in patients suffering from not better identified axonal polyneuropathy may be accompanied by an increased number of turns/s, similar to that observed in healthy VFs [16]. Also synkinesis can associate with an almost normal EMG pattern and number of turns/s [11]. Accordingly, its evaluation would require larger sample sizes than that featured by this preliminary study.

There are some limitations that could have affected the results of our study. In the first place, it was conducted monocentric and on a limited number of patients. In the second place, it is possible that although the turn number was calculated on the TA, it may include some values generated on the lateral cricoarytenoid (LCA) instead, due to the extreme proximity of the two muscles. Since our results are in line with previously published works, we expect that such potential error did not constitute a significant bias for our result analysis.

## Conclusions

Our study is one of the first attempts to compare the results of qualitative vs. semi-quantitative analysis applied to LEMG.

Our initial results on a limited sample of data sets seem to confirm that the implementation of turn calculation could become a useful tool to confirm volitional activity assessment and help discriminating UVFI due to dysfunctionalities of the thyroarytenoid muscle from those caused by glottic gap reduction due to mechanical causes such as stenosis.

Future studies in larger cohorts and including standard voice assessments and/or laryngostroboscopy should be considered confirming these results.
